# Discovery Genome-Wide Association Study of Body Composition in 4,386 Adults From the UK Biobank’s Pilot Imaging Enhancement Study

**DOI:** 10.3389/fendo.2021.692677

**Published:** 2021-06-22

**Authors:** Katherine M. Livingstone, Mun Hua Tan, Gavin Abbott, Rachel L. Duckham, Larry Croft, Joey Ward, Mark McEvoy, Michelle A. Keske, Christopher Austin, Steven J. Bowe

**Affiliations:** ^1^ Institute for Physical Activity and Nutrition, School of Exercise and Nutrition Sciences, Deakin University, Geelong, VIC, Australia; ^2^ Department of Microbiology and Immunology, Bio21 Institute, University of Melbourne, Melbourne, VIC, Australia; ^3^ Australian Institute for Musculoskeletal Science (AIMSS), The University of Melbourne and Western Health, St Albans, VIC, Australia; ^4^ School of Life and Environmental Sciences, Deakin Genomics Centre, Deakin University, Geelong, VIC, Australia; ^5^ Institute of Health and Wellbeing, University of Glasgow, Glasgow, United Kingdom; ^6^ La Trobe Rural Health School, College of Science, Health and Engineering, La Trobe University, Bendigo, VIC, Australia; ^7^ Deakin Biostatistics Unit, Deakin University, Geelong, VIC, Australia

**Keywords:** dual energy X-ray absorptiometry, genome-wide association study, loci, bone mass, fat mass, lean mass, body composition, genetics

## Abstract

Body composition (fat, skeletal muscle and bone mass) is an important determinant of overall health and risk of endocrine disorders such as type 2 diabetes and osteoporosis. Although diet and physical activity are strongly implicated, body composition is also heritable. We conducted a discovery genome-wide association study on 31 phenotypes from the three-compartment body composition model (fat, lean and bone mass) in a set of 4 386 individuals (n = 2 109 males, n = 2 294 females) from the UK Biobank pilot imaging enhancement program that underwent a dual energy X-ray absorptiometry (DXA) scan for assessment of body composition and genetic screening. From 6 137 607 imputed single nucleotide polymorphisms (SNPs) we identified 17 body composition loci (P<5.0 x 10-8). GWAS from the combined dataset identified four statistically significant SNPs (rs7592270, rs145972737, rs13212044, rs77772562). In sex-stratified GWAS, 10 male specific SNPs across all traits were identified and five female specific SNPs. Of the 17 SNPs, six were in or close to a gene where there was a plausible functional connection. Three SNPs (rs7592270, rs77772562 and rs7552312) were correlated with obesity phenotypes, one SNP (rs2236705) with lean phenotypes and two with bone mass phenotypes (rs112098641 and rs113380185). These results highlight candidate genes and biological pathways related to body composition, including glucose metabolism and estrogen regulation, that are of interest to replicate in future studies.

## Introduction

Body composition is implicated in the progression of many chronic diseases, including endocrine disorders such as type 2 diabetes (T2D) (1). The heritability of body composition is high, with total and regional distribution of fat, lean and bone mass governed by genetic susceptibility (2). The increasing availability of genetic data from large population-based cohorts provides an opportunity for Genome-Wide-Association Studies (GWAS) to identify causal Single Nucleotide Polymorphisms (SNPs) with multiple body composition phenotypes.

Most body composition GWAS research has focused on the genetics of body mass index (BMI) (2), waist and hip circumference (3) or singular compartments of body composition, such as lean or fat mass (4, 5). While previous GWAS have investigated the role of bone content of specific regions (6), few have investigated the genetics of total and regional bone mass. These studies have advanced understanding of the role and potential mechanisms of common genetic variations in BMI and body fat distribution, including sexual dimorphisms in the genetic regulation of these traits. Specifically, pathway analyses suggest that adipose tissue deposition and BMI are closely linked with insulin regulation and lipid biology, and thus share pathways with T2D and glycaemic traits (2, 3). These phenotypes have also been linked with skeletal growth processes (3), yet the common genetic variations in fat, lean and bone phenotypes remain unclear as they have not been investigation simultaneously. To our knowledge, no GWAS have investigated the genetics of all three body composition compartments simultaneously. Understanding the genetics of the three-compartment model of body composition will provide new insights into the shared heritability of and inter-connection between fat, lean and bone traits. Moreover, the majority of research has derived estimates of fat and lean mass from bio-electrical impedance analysis, which has inflexible hydration assumptions and is unable to assess bone mass (7). While dual energy X-ray absorptiometry (DXA) is the gold standard for assessing the three-compartment model of body composition, the high cost and exposure to radiation has largely precluded application in a large population cohort (8).

The availability of gold standard DXA data on 5 170 participants from the UK Biobank (UKB) pilot imaging enhancement program provided an opportunity to perform a discovery GWAS on the genetic determinants of fat, lean and bone body composition. Sex-stratified analyses were performed to identify SNPs that differed between males and females.

## Methods

UKB is a population cohort of 500 000 individuals aged 40 to 69 years living in the United Kingdom (9). Individuals identified from National Health Service patient registers were invited to one of 22 assessment centres between 2006 and 2020 to provide deep phenotyping and molecular data. UKB received approval from the National Information Governance Board for Health and Social Care and the National Health Service North West Centre for Research Ethics Committee (Ref: 11/NW/0382). All participants provided informed consent to participate. UKB imaging enhancement program will make DXA data available for 100 000 individuals by 2023 (10). Between 2014 to 2015, UKB undertook a pilot study in 5 170 participants. For the present study, complete case analysis was used. Participants were excluded if they had missing call rate of > 0.1, unusual level of heterozygosity, sex mismatch, sex chromosome aneuploidy, had missing data on sex, or were not of ‘White British’ descent.

Total and regional fat, lean and bone mass were determined from a total body GE-Lunar iDXA scan (GE Healthcare, Madison, Wisconsin, USA) and analyzed with Encore software (version 11.0) using standardized procedures. All scans were collected and analyzed by a trained radiographer. For the purpose of this study, 31 fat, lean and bone phenotypes were included (**Table 1**).

**Table 1:** Mean (SD) of body composition phenotypes in combined, female and male datasets.

Body composition phenotype	Combined (n=4 386)	Female (n=2 294)	Male (n=2 109)
Obesity-related			
Android fat mass (g)	2 456 (1229)	2 213 (1151)	2 715 (1257)
Gynoid fat mass (g)	4 166 (1544)	4 686 (1544)	3 596 (1327)
Arms fat mass (g)	2 664 (976)	2 926 (1045)	2 377 (802)
Legs fat mass (g)	7 744 (3084)	9 088 (3081)	6 278 (2318)
Trunk fat mass (g)	14 677 (6182)	14 001 (6033)	15 386 (6260)
VAT mass (g)^1^	1 225 (912)	781 (580)	1 703 (963)
VAT volume (cm^3^)^1^	1 298 (967)	828 (614)	1 805 (1021)
Lean-related			
Android lean mass (g)	3 488 (766)	2 911 (413)	4 115 (535)
Gynoid lean mass (g)	7 333 (1604)	6 123 (797)	8 649 (1170)
Arms lean mass (g)	5 381 (1594)	4 091 (638)	6 783 (1044)
Legs lean mass (g)	15 975 (3704)	13 279 (2043)	18 905 (2773)
Trunk lean mass (g)	22 866 (4443)	19 468 (2273)	26 556 (3080)
Bone-related			
Android bone mass (g)	49.3 (13.1)	42.5 (9.5)	56.6 (12.5)
Gynoid bone mass (g)	275 (67.5)	227 (37.5)	328 (52.0)
Arms bone mineral content (g)	365 (100)	283 (44.3)	453 (62.4)
Legs bone mineral content (g)	985 (237)	805 (124)	1 180 (165)
Trunk bone mineral content (g)	780 (203)	651 (131)	919 (172)
Ratios			
Arm fat: total fat	0.10 (0.02)	0.11 (0.02)	0.10 (0.01)
Leg fat: total fat	0.30 (0.07)	0.35 (0.06)	0.26 (0.04)
Trunk fat: total fat	0.56 (0.08)	0.51 (0.07)	0.60 (0.06)
Android fat: gynoid fat	0.59 (0.22)	0.46 (0.15)	0.74 (0.18)
Trunk fat: peripheral fat	1.45 (0.47)	1.16 (0.31)	1.77 (0.39)
Total			
Android mass (kg)	5.99 (1.72)	5.17 (1.40)	6.89 (1.58)
Gynoid mass (kg)	11.8 (2.29)	11.0 (2.12)	12.6 (2.20)
Arms mass (kg)	8.41 (1.95)	7.30 (1.54)	9.61 (1.61)
Legs mass (kg)	24.7 (4.91)	23.2 (4.71)	26.4 (4.56)
Trunk mass (kg)	38.3 (8.81)	34.1 (7.26)	42.9 (8.06)
Total bone mineral content (g)	2 644 (572)	2 229 (337)	3 094 (413)
Total fat mass (g)	25 967 (9383)	26 825 (9570)	25 003 (9072)
Total lean mass (g)	47 356 (9821)	39 702 (4760)	55 673 (6649)
Total mass (kg)	76.0 (15.4)	68.8 (13.0)	83.8 (13.9)

^1^VAT mass and volume were available in n = 4 336 (combined), 2 266 (female) and 2 088 (male). Visceral Adipose Tissue, VAT. Arm, leg and trunk fat ratio were calculated by dividing arms, legs and trunk fat mass (g) by total fat mass (g), respectively. We estimated android gynoid ratio by dividing android fat mass (g) by gynoid fat mass (g). Trunk peripheral ratio was estimated by dividing trunk fat mass (g) by the sum of arms and legs fat mass (g).

The March 2018 release of the imputed genetic data (analyzed from blood samples) from UK Biobank (downloaded 11 November 2019) was used (11). Genome-wide association analysis for each of the 31 phenotypes was performed on imputed SNPs with PLINK v2.00a3LM, using the generalized linear model suitable for quantitative phenotypes. In combined analysis, genotyping platform, genetic sex and the first four principal genetic components were set as covariates. Resulting polymorphisms with missing call rates of greater than 0.05, Hardy-Weinberg equilibrium p-values of less than 1 x 10^-6^, allele frequencies lower than 0.01 and/or imputation scores of less than 0.8 were excluded from the final set variants. The ‘estlambda’ function in GenABEL library was used to estimate genomic inflation factor of each GWAS analysis. SNP associations with p-values < 5.0 x 10^-8^ were considered statistically significant. Bonferroni correction was used to correct for multiple testing. Related individuals were identified based on kinship coefficient values and, for any pair of individuals with valid phenotypic values, one was excluded at random. The LD Score Regression was used to estimate heritability and correlations of phenotypes. Correlations were deemed strong at r>0.6 and significant at P>0.05. We cross-referenced lead SNPs from all independent signals in our analyses with the NHGRI-EBI catalog of published GWAS to determine whether fat, lean and bone-associated signals overlapped with previously identified associations from previous GWAS (GWAS Catalog—downloaded 9 July 2020). For SNPs identified at p-values < 5.0 x 10^-8^, SNPs were inspected to ascertain proximity to a coding gene and thus potential plausible functional connections. Only SNPs in or very close to a gene where there was a plausible functional connection were further discussed.

## Results

A total of 4 386 participants (n=2 109 male, n=2 294 female) were included (**Figure S1**). Mean age at recruitment was 55.9 (SD 7.5). Mean (SD) of obesity-, lean-, and bone-related body composition phenotypes, as well as body composition ratios, are presented in **Table 1**. The genomic inflation factors for the combined and sex-stratified GWAS are presented in **Table S1**. For the combined dataset, genomic inflation factors ranged between 1.010 (SE 2.12 X 10^-6^) to 1.046 (SE 4.52 X 10^-6^). Heritability estimates for body composition phenotypes in the combined dataset are shown in **Table S2** and ranged between 0.08 (SE 0.12) to 0.51 (SE 0.14). Correlations for body composition phenotypes are shown in **Table S3**. With the exception of leg fat ratio, android gynoid ratio, trunk bone mineral content, trunk peripheral ratio and Visceral Adipose Tissue (VAT) mass and volume, the majority of the 31 body composition phenotypes were strongly and significantly positively correlated with most other phenotypes.

**Figure 1:**
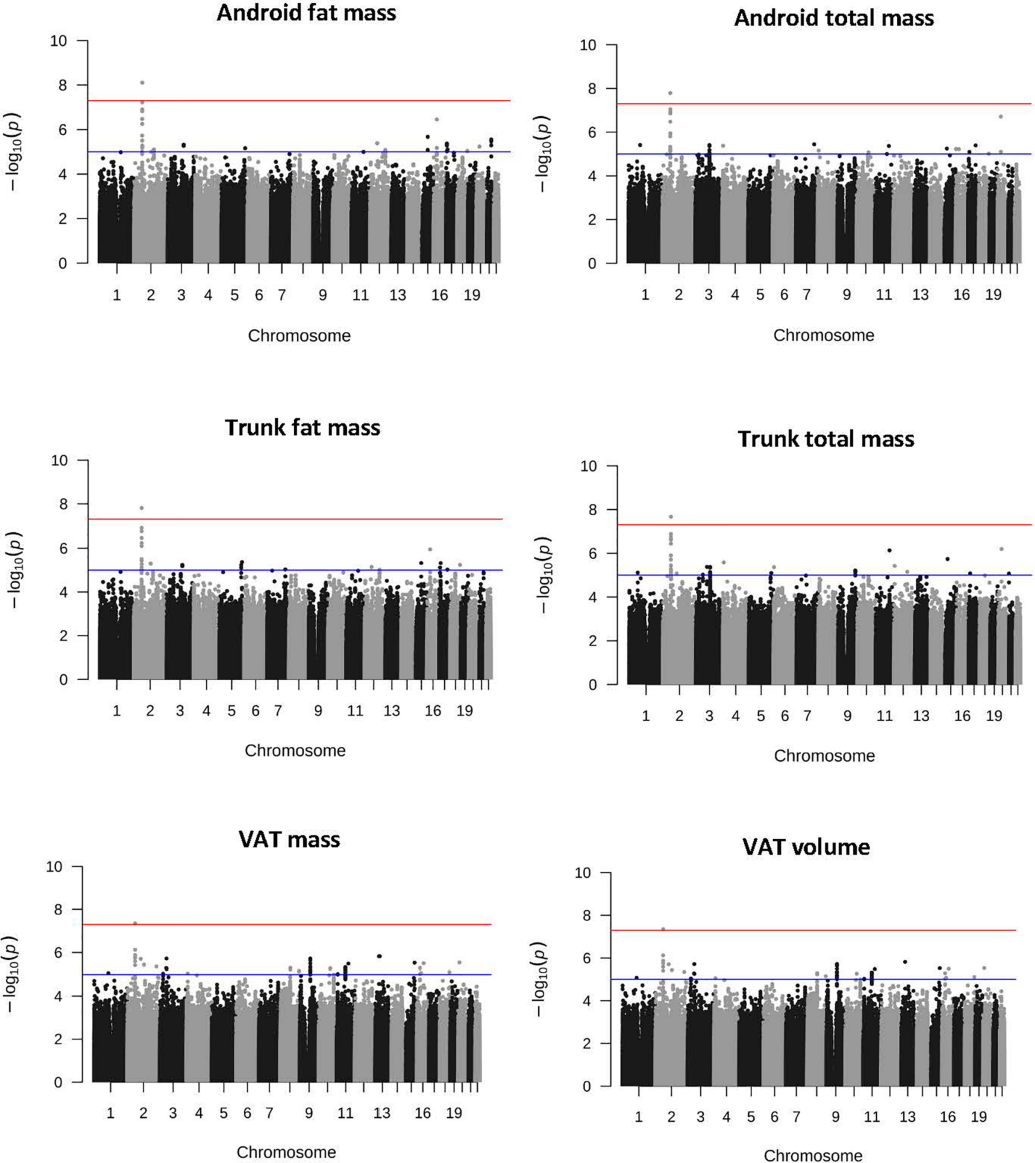
Manhattan plot of obesity-related SNP rs7592270 statistically significant above line at p-value 5.0 x 10^-8^ in the combined (male and female) GWAS (n = 4 386; VAT, n = 4 336) VAT, visceral adipose tissue.

As shown in **Table 2**, from 6 137 607 imputed SNPs, we identified 17 body composition loci (P<5.0 x 10-8). GWAS from the combined dataset identified four statistically significant SNPs (rs7592270, rs145972737, rs13212044, rs77772562; **Figure S2**). In sex-stratified GWAS, 10 male specific SNPs across all traits were identified and five female specific SNPs (**Table 1**). Of these, six SNPs were in or close to a gene where there was a plausible functional connection (rs7592270, rs77772562, rs7552312, rs2236705, rs112098641 and rs113380185; **Figure 1**, **Table 1**, **Figures S3** and **S4**). Three SNPs were associated with obesity phenotypes in the combined dataset. SNP rs7592270 was correlated with female android fat mass, android total mass, trunk total mass, VAT mass and VAT volume, SNP rs7552312 was correlated with trunk total fat ratio and SNP rs77772562 was correlated with the trunk peripheral ratio. One SNP rs2236705 was correlated with lean phenotypes and two SNPs (rs112098641 and rs113380185) were correlated with bone phenotypes. In sex-stratified datasets, SNP rs2236705 was correlated with female lean leg mass, SNP rs112098641 was associated with female gynoid bone mass and SNP rs113380185 was correlated with male android bone mass (**Table 1**, **Figures S3** and **S4**).

**Table 2:** List of SNPs in combined, female and male datasets significant at p< 5.0 x 10^-8^.

	Chr	Position	SNP ID	Ref allele	Alt allele	Eff allele	Annotation	Combined (n=4 386)		Female (n=2 294)			Male (n=2 109)	
								Beta	P-value		Beta	P-value	Beta	P-value
**Obesity-related phenotypes**														
Android fat mass	2	58799070	rs7592270	C	T	T	*LINC01122(0)*	401.91	7.84 x 10^-9^		–	–	–	–
Gynoid fat mass	6	17429040	rs113380185	C	T	T	*CAP2(0)*	–	–		–	–	959.03	1.58 x 10^-8^
	13	72584929	13:72584929_AAC_A	AAC	A	A	.	–	–		–	–	759.52	1.89 x 10^-8^
Arms fat mass	12	11727655	rs117686994	T	G	G	.	–	–		662.11	1.36 x 10^-8^	–	–
Legs fat mass	7	15845439	rs143707182	C	G	G	.	–	–		–	–	1782.6	2.48 x 10^-8^
	13	27409628	rs138014219	A	T	T	.	–	–		–	–	2194.3	2.96 x 10^-8^
	13	72584929	13:72584929_AAC_A	AAC	A	A	.	–	–		–	–	1333.3	1.59 x 10^-8^
Trunk fat mass	2	58799070	rs7592270	C	T	T	*LINC01122(0)*	2011.4	1.56 x 10^-8^		–	–	–	–
VAT mass	2	58799070	rs7592270	C	T	T	*LINC01122(0)*	251.04	4.49 x 10^-8^		–	–	–	–
VAT volume	2	58799070	rs7592270	C	T	T	*LINC01122(0)*	266.09	4.49 x 10^-8^		–	–	–	–
**Lean-related phenotypes**														
Gynoid lean mass	3	32262434	rs145972737	G	A	A	.	510.27	3.39 x 10^-8^		–	–	910.02	4.64 x 10^-9^
Legs lean mass	21	43732828	rs2236705	C	A	A	*TFF3(0)*	–	–		465.92	2.55 x 10^-8^	–	–
Trunk lean mass	6	127025661	rs13212044	G	T	T	.	-353.86	4.27 x 10^-8^		–	–	–	–
**Bone-related phenotypes**														
Android bone mass	6	33901724	rs138986597	G	A	A	.	–	–		–	–	11.23	8.69 x 10^-9^
Gynoid bone mass	9	114333327	rs72748040	T	A	A	*PTGR1(0) ZNF483(0)*	–	–		–	–	26.29	3.20 x 10^-8^
	14	27073424	rs112098641	T	G	G	*NOVA1(+6.464kb)*	–	–		30.62	4.54 x 10^-8^	–	–
Arms bone mineral content	17	39911373	rs55634776	C	T	T	*JUP(0)*	–	–		–	–	39.77	1.17 x 10^-8^
Trunk bone mineral content	14	27073424	rs112098641	T	G	G	*NOVA1(+6.464kb)*	–	–		107.64	4.12 x 10^-8^	–	–
**Total**														
Android total mass	2	58799070	rs7592270	C	T	T	*LINC01122(0)*	0.49	1.63 x 10^-8^		–	–	–	–
Arms total mass	3	11244120	rs35932350	A	T	T	*HRH1(0)*	–	–		0.65	2.15 x 10^-8^	–	–
	12	11727655	rs117686994	T	G	G	.	–	–		0.98	1.05 x 10^-8^	–	–
Legs total mass	3	8684177	rs164955	A	C	A	*SSUH2(0)*	–	–		2.06	7.04 x 10^-9^	–	–
	21	43732828	rs2236705	C	A	A	*TFF3(0)*	–	–		1.13	4.23 x 10^-9^	–	–
Trunk total mass	2	58799070	rs7592270	C	T	T	*LINC01122(0)*	2.48	2.13 x 10^-8^		–	–	–	–
**Ratios**														
Trunk: total fat	1	46657220	rs7552312	C	T	T	*POMGNT1(0) TSPAN1(+5.586kb)*	–	–		–	–	-0.01	4.19 x 10^-8^
Android: gynoid	17	6289983	rs1567843	G	C	G	.	–	–		–	–	-0.05	3.86 x 10^-8^
Trunk: peripheral	10	80755929	rs77772562	C	A	A	*ZMIZ1-AS1(0)*	0.18	4.97 x 10^-8^		–	–	–	–

VAT (visceral adipose tissue) mass and volume were available in n=4 336 (combined), 2 266 (female) and 2 088 (male). A positive beta indicates that as the number of copies of the minor allele increases the outcome increases by beta*number of copies of the minor frequency allele, while a negative beta indicates that the outcome variable decreases by beta*number of copies of the minor frequency allele. Trunk fat ratio was calculated by dividing trunk fat mass (g) by total fat mass (g). We estimated android gynoid ratio by dividing android fat mass (g) by gynoid fat mass (g). Trunk peripheral ratio was estimated by dividing trunk fat mass (g) by the sum of arms and legs fat mass (g). VAT, visceral adipose tissue; Ref, reference; Alt, alternative.

## Discussion

The aim of this discovery GWAS was to provide preliminary evidence for the role of genetics in the three-compartment model of body composition. From the 6 137 607 imputed SNPs and 31 body composition phenotypes investigated, our main findings were that 17 SNPs were significantly associated with either fat, lean or bone phenotypes. Of these, six SNPs were linked to genes with known functional outcomes and thus help to explain the physiological mechanisms leading to body composition. These preliminary findings support the need for clinicians to consider the inter-connection between fat, lean and bone mass and provide new insights into biological pathways, including glucose metabolism and estrogen regulation, that will inform future research aimed at understanding the complex biology of body composition.

We identified three SNPs related to obesity that have been previously reported to be associated with risk of T2D (12, 13). Thus, these SNPs may exert their effects at increasing T2D risk by modifying body composition (specifically fat) and by altering glucose and insulin pathways (3). More specifically, SNP rs7592270 was correlated with female android fat mass, android total mass, trunk total mass, VAT mass and volume, all of which are indicators of harmful accumulation of central regional body fat often associated with increased risk of T2D (12). SNP rs7592270 is found in the noncoding gene *LINC01122*, which is correlated with anthropometric extremes and is near SNPs correlated to obesity class I (rs929641) and being overweight (rs887912) (14). SNPs rs7592270, rs929641 and rs887912 were in or very close to *LINC01122*. This SNP is found in the antisense noncoding gene *ZMIZ1-AS1*, sharing a bidirectional promoter with *ZMIZ1*. *ZMIZ1*, a transcription factor regulator, is involved in glucose regulation and diabetes (15). SNP rs7552312 was correlated with male trunk fat mass and is found in the *POMGNT1* gene. Mutations in this gene are also associated with T2D (13), which provides another link between obesity and risk of T2D. T2D is often characterised by higher fat and lower skeletal muscle mass, increasing risk of inadequate glucose metabolism, while excess fat mass distribution within the central region may increase fat infiltration within the muscle and risk of insulin resistance (16), further supporting that these obesogenic SNPs are also linked to T2D risk.

We observed that three SNPs were related to lean and bone mass phenotypes and are likely to impact body composition *via* estrogen pathways. SNP rs2236705 was correlated with female lean leg mass. This SNP is found in the intron of the *TFF3* gene, which is involved in skeletal metabolism, is estrogen regulated and regulates glucose metabolism (17). SNP rs112098641 is associated with female gynoid bone mass. The SNP is 6kb upstream from the *NOVA1* gene in the intron of a lncRNA gene, which is estrogenically regulated (18). Moreover, estrogen plays a role in the development of lean tissue mass and bone mass regulation in adulthood (19). Estrogen acts through two receptors – the alpha and beta receptors – which are important in lean and bone metabolism, respectively. Through these receptors, estrogen protects bone metabolism by regulating the survival of osteoclasts, if circulating levels of estrogen are low, bone mass will be lost, such as that observed in post-menopausal women (19). Similarly, the estrogen-receptor alpha located on skeletal muscle may activate signalling pathways, such as Insulin-like Growth Factor-1, that mediates skeletal metabolism (20). SNP rs113380185, found in the *CAP2* gene, was correlated with male android bone mass and has been associated with human height (21) (**Table S1**, **Figures S3** and **S4**).

We acknowledge some limitations. Our findings are not generalizable to non-white populations. Our sample size is relatively small and we are likely underpowered to detect variants with small effect sizes. Nonetheless, these findings are from a pilot study and are designed to be replicated and to inform future research in the full 100 000 participants from the UKB imaging enhancement programme. Strengths of this study include the investigation of 31 phenotypes from the three-compartment model of body composition. These have been largely under-researched due to the high cost and participant burden of collecting DXA measures.

The present study identified six loci that help explain the physiological mechanisms leading to body composition. These preliminary findings support the connection between fat, lean and bone mass and the need for these compartments of body composition to be considered concurrently by clinicians. The biological pathways highlighted by this study, including glucose metabolism and estrogen regulation, will inform future research aimed at understanding the complex biology of body composition. Potential candidate genes, such as *LINC01122* and *POMGNT1*, are of interest to investigate in future studies, including the full UKB imaging enhancement programme dataset when it becomes available.

## Data Availability Statement

The data analyzed in this study is subject to the following licenses/restrictions: The UK Biobank data are available on application to the UK Biobank (www.ukbiobank.ac.uk/). Requests to access these datasets should be directed to www.ukbiobank.ac.uk/.

## Ethics Statement

UK Biobank received ethical approval from the National Information Governance Board for Health and Social Care and the National Health Service North West Centre for Research Ethics Committee (reference 13/NW/0382). All participants provided informed consent to participate. The present analyses were conducted under UK Biobank application number 34894. The patients/participants provided their written informed consent to participate in this study.

## Author Contributions

KL, JW, RD, MM, and SB designed the analysis. MT, GA, SB, JW, and LC conducted the statistical analysis. KL, MT, MK, and SB drafted the manuscript. All authors contributed to the article and approved the submitted version.

## Funding

KL is supported by a National Health and Medical Research Council Emerging Leadership Investigator Grant (APP1173803). JW is funded by the Lister Prize Fellowship (173096). The funding source had no role in the design or conduct of the study; collection, management, analysis, and interpretation of the data; or preparation, review, or approval of the manuscript.

## Conflict of Interest

The authors declare that the research was conducted in the absence of any commercial or financial relationships that could be construed as a potential conflict of interest.
