# Supplementary material for: Discovery Genome-Wide Association Study of Body Composition in 4,386 Adults From the UK Biobank’s Pilot Imaging Enhancement Study
Source: Front Endocrinol (Lausanne). 2021 Jun 22;12:692677. doi: 10.3389/fendo.2021.692677 (PMC8259458; doi:10.3389/fendo.2021.692677)
Supplement: Supplementary file 1 [file DataSheet_1.docx]

**Table S1** Genomic inflation factors combined and by sex

| **Body composition phenotype** | **Combined (n=4386)** | | **Female (n=2,294)** | | **Male (n=2,109)** | |
| --- | --- | --- | --- | --- | --- | --- |
|  | λ | **SE** | λ | **SE** | λ | **SE** |
| **Obesity-related** |  |  |  |  |  |  |
| Android fat mass | 1.013 | 3.55 X 10^-6^ | 1.005 | 1.92 X 10^-6^ | 1.017 | 3.02 X 10^-6^ |
| Gynoid fat mass | 1.019 | 2.99 X 10^-6^ | 1.005 | 1.94 X 10^-6^ | 1.023 | 5.93 X 10^-6^ |
| Arms fat mass | 1.014 | 1.86 X 10^-6^ | 1.013 | 2.48 X 10^-6^ | 1.016 | 7.36 X 10^-6^ |
| Legs fat mass | 1.017 | 1.80 X 10^-6^ | 1.002 | 9.47 X 10^-7^ | 1.025 | 6.76 X 10^-6^ |
| Trunk fat mass | 1.011 | 4.01 X 10^-6^ | 1.006 | 2.15 X 10^-6^ | 1.015 | 2.53 X 10^-6^ |
| VAT mass^1^ | 1.015 | 1.46 X 10^-6^ | 1.009 | 1.30 X 10^-6^ | 1.016 | 3.46 X 10^-6^ |
| VAT volume ^1^ | 1.015 | 1.46 X 10^-6^ | 1.009 | 1.29 X 10^-6^ | 1.016 | 3.46 X 10^-6^ |
| **Lean-related** |  |  |  |  |  |  |
| Android lean mass | 1.040 | 5.76 X 10^-6^ | 1.026 | 2.09 X 10^-6^ | 1.018 | 4.33 X 10^-6^ |
| Gynoid lean mass | 1.039 | 4.56 X 10^-6^ | 1.025 | 1.26 X 10^-6^ | 1.015 | 2.35 X 10^-6^ |
| Arms lean mass | 1.025 | 3.55 X 10^-6^ | 1.019 | 5.59 X 10^-6^ | 1.012 | 1.99 X 10^-6^ |
| Legs lean mass | 1.036 | 3.95 X 10^-6^ | 1.018 | 3.85 X 10^-6^ | 1.018 | 3.17 X 10^-6^ |
| Trunk lean mass | 1.046 | 4.52 X 10^-6^ | 1.026 | 1.71 X 10^-6^ | 1.018 | 2.78 X 10^-6^ |
| **Bone-related** |  |  |  |  |  |  |
| Android bone mass | 1.035 | 1.62 X 10^-6^ | 1.015 | 2.03 X 10^-6^ | 1.021 | 1.95 X 10^-6^ |
| Gynoid bone mass | 1.039 | 2.88 X 10^-6^ | 1.022 | 3.65 X 10^-6^ | 1.019 | 5.17 X 10^-6^ |
| Arms bone mineral content | 1.030 | 3.52 X 10^-6^ | 1.020 | 4.74 X 10^-6^ | 1.015 | 1.73 X 10^-6^ |
| Legs bone mineral content | 1.032 | 3.31 X 10^-6^ | 1.019 | 3.83 X 10^-6^ | 1.016 | 2.00 X 10^-6^ |
| Trunk bone mineral content | 1.042 | 3.15 X 10^-6^ | 1.022 | 4.24 X 10^-6^ | 1.021 | 4.45 X 10^-6^ |
| **Ratios** |  |  |  |  |  |  |
| Arm fat: total fat | 1.016 | 3.22 X 10^-6^ | 1.014 | 2.78 X 10^-6^ | 1.002 | 2.48 X 10^-6^ |
| Leg fat: total fat | 1.017 | 2.34 X 10^-6^ | 1.008 | 2.73 X 10^-6^ | 1.011 | 2.32 X 10^-6^ |
| Trunk fat: total fat | 1.010 | 2.12 X 10^-6^ | 1.005 | 3.26 X 10^-6^ | 1.008 | 1.78 X 10^-6^ |
| Android fat: gynoid fat | 1.020 | 3.86 X 10^-6^ | 1.011 | 1.85 X 10^-6^ | 1.016 | 3.18 X 10^-6^ |
| Trunk fat: peripheral fat | 1.015 | 1.42 X 10^-6^ | 1.006 | 3.33 X 10^-6^ | 1.010 | 1.88 X 10^-6^ |
| **Total** |  |  |  |  |  |  |
| Android mass | 1.021 | 3.12 X 10^-6^ | 1.012 | 1.86 X 10^-6^ | 1.020 | 2.03 X 10^-6^ |
| Gynoid mass | 1.028 | 1.81 X 10^-6^ | 1.012 | 2.40 X 10^-6^ | 1.022 | 4.70 X 10^-6^ |
| Arms mass | 1.020 | 4.08 X 10^-6^ | 1.016 | 3.14 X 10^-6^ | 1.012 | 5.28 X 10^-6^ |
| Legs mass | 1.028 | 2.56 X 10^-6^ | 1.011 | 2.89 X 10^-6^ | 1.023 | 4.30 X 10^-6^ |
| Trunk mass | 1.021 | 2.81 X 10^-6^ | 1.014 | 2.22 X 10^-6^ | 1.017 | 1.65 X 10^-6^ |
| Total bone mineral content | 1.039 | 4.39 X 10^-6^ | 1.024 | 3.61 X 10^-6^ | 1.018 | 2.28 X 10^-6^ |
| Total fat mass | 1.011 | 4.32 X 10^-6^ | 1.005 | 1.69 X 10^-6^ | 1.017 | 2.75 X 10^-6^ |
| Total lean mass | 1.043 | 4.50 X 10^-6^ | 1.025 | 3.00 X 10^-6^ | 1.017 | 3.97 X 10^-6^ |
| Total mass | 1.024 | 1.85 X 10^-6^ | 1.013 | 2.45 X 10^-6^ | 1.019 | 3.22 X 10^-6^ |

1, VAT (visceral adipose tissue) mass and volume were available in n=4 336 (combined), 2 266 (female) and 2 088 (male).

**Table S2** Heritability estimates determined using LD Score regression

| **Trait**^1^ | **h^2^ (se)** |
| --- | --- |
| **Obesity-related** |  |
| VAT mass^2^ | 0.08 (0.12) |
| VAT volume^2^ | 0.08 (0.12) |
| **Lean-related** |  |
| Android lean mass | 0.38 (0.14) |
| Gynoid lean mass | 0.40 (0.14) |
| Arms lean mass | 0.25 (0.14) |
| Trunk lean mass | 0.51 (0.14) |
| **Bone-related** |  |
| Android bone mass | 0.49 (0.15) |
| Gynoid bone mass | 0.48 (0.15) |
| Arms bone mineral content | 0.37 (0.15) |
| Legs bone mineral content | 0.39 (0.14) |
| Trunk bone mineral content | 0.47 (0.16) |
| **Ratios** |  |
| Leg fat: total fat | 0.15 (0.12) |
| Android fat: gynoid fat | 0.17 (0.12) |
| Trunk fat: peripheral fat | 0.10 (0.12) |
| **Total** |  |
| Android mass | 0.23 (0.12) |
| Gynoid mass | 0.33 (0.13) |
| Arms mass | 0.27 (0.12) |
| Legs mass | 0.34 (0.12) |
| Trunk mass | 0.25 (0.13) |
| Total bone mineral content | 0.50 (0.16) |
| Total mass | 0.30 (0.12) |

1, Estimates for 10 traits were not reported as the median beta were just outside of this boundary and because the estimates came out as less than zero

2, VAT (visceral adipose tissue) mass and volume were available in n=4 336 (combined)

**Table S3** Correlations between body composition phenotypes determined using LD Score regression

| \ | **Android bone mass** | **Android gynoid ratio** | **Android lean mass** | **Android total mass** | **Arms BMC** | **Arms lean mass** | **Arms total mass** | **Gynoid bone mass** | **Gynoid lean mass** | **Gynoid total mass** | **Leg fat ratio** | **Legs BMC** | **Legs total mass** | **Total BMC** | **Total mass** | **Trunk BMC** | **Trunk lean mass** | **Trunk peripheral ratio** | **Trunk total mass** | **VAT mass** | **VAT volume** |
| --- | --- | --- | --- | --- | --- | --- | --- | --- | --- | --- | --- | --- | --- | --- | --- | --- | --- | --- | --- | --- | --- |
| **Android bone mass** | 1 | *NA* | *NA* | *NA* | *NA* | *NA* | *NA* | *NA* | *NA* | *NA* | *NA* | *NA* | *NA* | *NA* | *NA* | *NA* | *NA* | *NA* | *NA* | *NA* | *NA* |
| **Android gynoid ratio** | -0.15 (0.36), 0.67 | 1 | *NA* | *NA* | *NA* | *NA* | *NA* | *NA* | *NA* | *NA* | *NA* | *NA* | *NA* | *NA* | *NA* | *NA* | *NA* | *NA* | *NA* | *NA* | *NA* |
| **Android lean mass** | 0.49  (0.19),  0.012 | -0.19  (0.37), 0.61 | 1 | *NA* | *NA* | *NA* | *NA* | *NA* | *NA* | *NA* | *NA* | *NA* | *NA* | *NA* | *NA* | *NA* | *NA* | *NA* | *NA* | *NA* | *NA* |
| **Android total mass** | 0.33  (0.28), 0.23 | -0.05  (0.55), 0.92 | 0.88  (0.15), <0.001 | 1 | *NA* | *NA* | *NA* | *NA* | *NA* | *NA* | *NA* | *NA* | *NA* | *NA* | *NA* | *NA* | *NA* | *NA* | *NA* | *NA* | *NA* |
| **Arms BMC** | 0.86  (0.12), <0.001 | 0.01  (0.34), 0.97 | 0.68  (0.18), 0.001 | 0.39  (0.29), 0.17 | 1 | *NA* | *NA* | *NA* | *NA* | *NA* | *NA* | *NA* | *NA* | *NA* | *NA* | *NA* | *NA* | *NA* | *NA* | *NA* | *NA* |
| **Arms lean mass** | 0.61  (0.24), 0.013 | -0.19  (0.42), 0.65 | 0.83  (0.13), <0.001 | 0.68  (0.21), 0.001 | 0.92  (0.16), <0.001 | 1 | *NA* | *NA* | *NA* | *NA* | *NA* | *NA* | *NA* | *NA* | *NA* | *NA* | *NA* | *NA* | *NA* | *NA* | *NA* |
| **Arms total mass** | 0.55  (0.24), 0.023 | -0.06  (0.43), 0.88 | 0.84  (0.14), <0.001 | 0.89  (0.09), <0.001 | 0.72  (0.20), 0.001 | 0.90  (0.09), <0.001 | 1 | *NA* | *NA* | *NA* | *NA* | *NA* | *NA* | *NA* | *NA* | *NA* | *NA* | *NA* | *NA* | *NA* | *NA* |
| **Gynoid bone mass** | 0.86  (0.08), <0.001 | -0.06  (0.32), 0.84 | 0.91  (0.13), <0.001 | 0.61  (0.23), 0.001 | 0.83  (0.09), <0.001 | 0.91  (0.18), <0.001 | 0.74  (0.18), <0.001 | 1 | *NA* | *NA* | *NA* | *NA* | *NA* | *NA* | *NA* | *NA* | *NA* | *NA* | *NA* | *NA* | *NA* |
| **Gynoid lean mass** | 0.59  (0.18), 0.001 | -0.41  (0.42), 0.33 | 0.97  (0.06), <0.001 | 0.75  (0.17), <0.001 | 0.61  (0.17), 0.001 | 0.87  (0.09), <0.001 | 0.73  (0.16), <0.001 | 0.81  (0.10), <0.001 | 1 | *NA* | *NA* | *NA* | *NA* | *NA* | *NA* | *NA* | *NA* | *NA* | *NA* | *NA* | *NA* |
| **Gynoid total mass** | 0.52  (0.21), 0.014 | -0.63  (0.54), 0.25 | 0.97  (0.09), <0.001 | 0.88  (0.08), <0.001 | 0.44  (0.23), 0.06 | 0.80  (0.15), <0.001 | 0.82  (0.10), <0.001 | 0.65  (0.16), <0.001 | 0.89  (0.07), <0.001 | 1 | *NA* | *NA* | *NA* | *NA* | *NA* | *NA* | *NA* | *NA* | *NA* | *NA* | *NA* |
| **Leg fat ratio** | 0.10  (0.36), 0.78 | -0.69  (0.25), 0.001 | 0.28  (0.40), 0.49 | 0.43  (0.71), 0.54 | 0.01  (0.38), 0.99 | 0.34  (0.49), 0.49 | 0.24  (0.51), 0.64 | -0.01  (0.33), 0.99 | 0.58  (0.48), 0.23 | 0.85  (0.60), 0.15 | 1 | *NA* | *NA* | *NA* | *NA* | *NA* | *NA* | *NA* | *NA* | *NA* | *NA* |
| **Legs BMC** | 0.83  (0.11), <0.001 | 0.045 (0.34), 0.89 | 0.85  (0.14), <0.001 | 0.65  (0.26), 0.011 | 0.86  (0.07), <0.001 | 0.90  (0.19), <0.001 | 0.72  (0.20), 0.001 | 0.93  (0.04), <0.001 | 0.76  (0.12), <0.001 | 0.62  (0.19), 0.001 | -0.10  (0.37), 0.80 | 1 | *NA* | *NA* | *NA* | *NA* | *NA* | *NA* | *NA* | *NA* | *NA* |
| **Legs total mass** | 0.43  (0.22), 0.05 | -0.40  (0.45), 0.37 | 0.91  (0.10), <0.001 | 0.95  (0.09), <0.001 | 0.41  (0.23), 0.08 | 0.83  (0.14), <0.001 | 0.85  (0.09), <0.001 | 0.58  (0.17), 0.001 | 0.85  (0.10), <0.001 | 0.98  (0.02), 0.001 | 0.69  (0.50), 0.17 | 0.59  (0.18), 0.001 | 1 | *NA* | *NA* | *NA* | *NA* | *NA* | *NA* | *NA* | *NA* |
| **Total BMC** | 0.86  (0.07), <0.001 | -0.06  (0.32), 0.85 | 0.75  (0.15), <0.001 | 0.56  (0.23), 0.01 | 0.91  (0.05), <0.001 | 0.89  (0.18), <0.001 | 0.76  (0.17), <0.001 | 0.96  (0.03), <0.001 | 0.72  (0.12), <0.001 | 0.64  (0.16), <0.001 | 0.06  (0.34), 0.86 | 0.97  (0.03), <0.001 | 0.61  (0.16), <0.001 | 1 | *NA* | *NA* | *NA* | *NA* | *NA* | *NA* | *NA* |
| **Total mass** | 0.43  (0.24), 0.07 | -0.26  (0.50), 0.60 | 0.96  (0.10), <0.001 | 0.97  (0.03), <0.001 | 0.48  (0.24), 0.04 | 0.83  (0.13), <0.001 | 0.92  (0.05), <0.001 | 0.70  (0.17), <0.001 | 0.84  (0.10), <0.001 | 0.94  (0.03), <0.001 | 0.50  (0.59), 0.39 | 0.70  (0.19), 0.001 | 0.98  (0.03), <0.001 | 0.67  (0.17), <0.001 | 1 | *NA* | *NA* | *NA* | *NA* | *NA* | *NA* |
| **Trunk BMC** | 0.93  (0.05), <0.001 | -0.09  (0.37), 0.81 | 0.78  (0.16), <0.001 | 0.57  (0.22), 0.001 | 0.87  (0.09), <0.001 | 0.83  (0.18), <0.001 | 0.78  (0.18), <0.001 | 0.97  (0.04), <0.001 | 0.73  (0.13), <0.001 | 0.66  (0.16), <0.001 | 0.12  (0.37), 0.75 | 0.95  (0.07), <0.001 | 0.63  (0.16), 0.001 | 0.99  (0.02), <0.001 | 0.67  (0.17), <0.001 | 1 | *NA* | *NA* | *NA* | *NA* | *NA* |
| **Trunk lean mass** | 0.49  (0.18), 0.006 | -0.29  (0.34), 0.40 | 0.99  (0.03), <0.001 | 0.77  (0.18), <0.001 | 0.67  (0.15), <0.001 | 0.89  (0.09), <0.001 | 0.81  (0.16), <0.001 | 0.92  (0.10), <0.001 | 0.96  (0.04), <0.001 | 0.89  (0.09), <0.001 | 0.27  (0.37), 0.47 | 0.88  (0.10), <0.001 | 0.84  (0.11), <0.001 | 0.77  (0.11), <0.001 | 0.88  (0.11), <0.001 | 0.75  (0.13), <0.001 | 1 | *NA* | *NA* | *NA* | *NA* |
| **Trunk peripheral ratio** | -0.06  (0.43), 0.89 | 0.92  (0.13), <0.001 | 0.06  (0.43), 0.88 | 0.01  (0.65), 0.98 | 0.19  (0.45), 0.67 | 0.03  (0.50), 0.95 | 0.14  (0.50), 0.78 | 0.27  (0.36), 0.46 | -0.14  (0.47), 0.77 | -0.46  (0.67), 0.49 | -0.88  (0.13), <0.001 | 0.44  (0.47), 0.35 | -0.25  (0.55), 0.65 | 0.19  (0.38), 0.62 | -0.11  (0.59), 0.85 | 0.03  (0.42), 0.94 | -0.06  (0.40), 0.88 | 1 | *NA* | *NA* | *NA* |
| **Trunk total mass** | 0.39  (0.26), 0.14 | -0.23  (0.58),  0.70 | 0.99  (0.14), <0.001 | 0.98  (0.02), <0.001 | 0.45  (0.27), 0.10 | 0.80  (0.17), <0.001 | 0.93  (0.07), <0.001 | 0.73  (0.20), 0.001 | 0.84  (0.14), <0.001 | 0.91  (0.06), <0.001 | 0.46  (0.68), 0.51 | 0.76  (0.22), 0.001 | 0.97  (0.07), <0.001 | 0.66  (0.20), 0.001 | 0.99  (0.01), <0.001 | 0.64  (0.19), 0.001 | 0.90  (0.15), <0.001 | -0.11  (0.69),  0.88 | 1 | *NA* | *NA* |
| **VAT mass** | -0.06  (0.52), 0.91 | 0.07  (0.87),  0.94 | 0.68  (0.49), 0.16 | 0.92  (0.25), 0.001 | 0.55  (0.56), 0.32 | 0.77  (0.53), 0.15 | 1.00  (0.54), 0.042 | 0.76  (0.58), 0.19 | 0.67  (0.49), 0.17 | 0.71  (0.39), 0.07 | 0.06  (0.93), 0.96 | 0.76  (0.63), 0.23 | 0.77  (0.43), 0.07 | 0.66  (0.52), 0.20 | 0.88  (0.34), 0.010 | 0.53  (0.42), 0.21 | 0.55  (0.46), 0.24 | 0.13  (1.01),  0.90 | 0.86  (0.27), 0.001 | 1 | *NA* |
| **VAT volume** | -0.06  (0.52), 0.91 | 0.07  (0.87),  0.94 | 0.68  (0.49), 0.16 | 0.92  (0.25), 0.001 | 0.55  (0.56), 0.32 | 0.77  (0.53), 0.15 | 1.00  (0.54), 0.042 | 0.76  (0.58), 0.19 | 0.67  (0.49), 0.17 | 0.71  (0.39), 0.07 | 0.05  (0.93), 0.96 | 0.76  (0.63), 0.23 | 0.77  (0.43), 0.07 | 0.66  (0.52), 0.20 | 0.88  (0.34), 0.010 | 0.53  (0.42), 0.21 | 0.55  (0.46), 0.24 | 0.13  (1.01),  0.90 | 0.86  (0.27), 0.001 | 1 | 1 |

BMC, bone mineral content; VAT, visceral adipose tissue; Values represent regression coefficient (SE), P value. Grey cells represent statistically significant at P>0.05.

UK Biobank participants

n=502 536

EXCLUDED (n = 497 366)

- Not included in UK Biobank pilot imaging enrichment programme n=497 366

Pilot DXA data available

n=5 170

UNUSABLE GENETIC DATA (n=784)

- Did not pass genetic quality control, n=53
- Exclusion due to relatedness, n=40
- Missing data on sex, n=691

DATA AVAILABLE FOR ANALYSIS

N=4 386

n=2 109 males*

n= 2 294 females*

**Figure S1.** Flow diagram of participants in the UK Biobank

DXA, dual-energy X-ray Absorptiometry

* For sex-stratified analyses, exclusion due to relatedness was performed after segregation of dataset by sex.

**
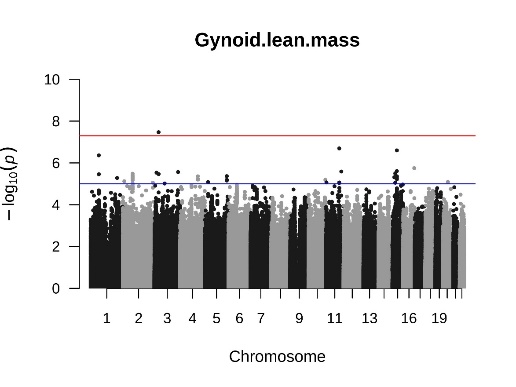

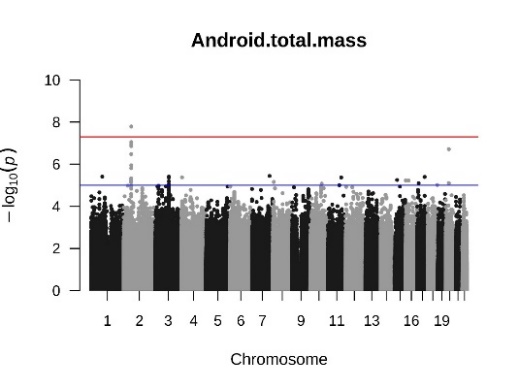

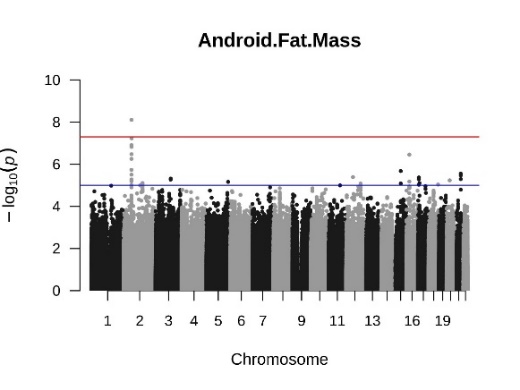
rs7592270 rs7592270 rs145972737**

**
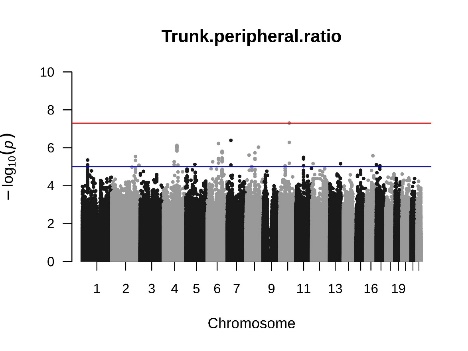

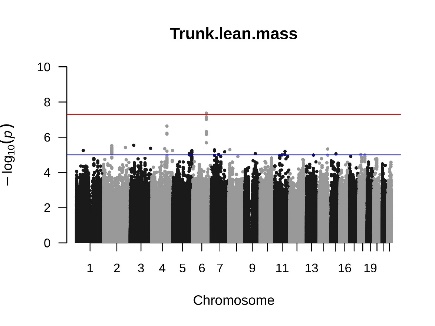

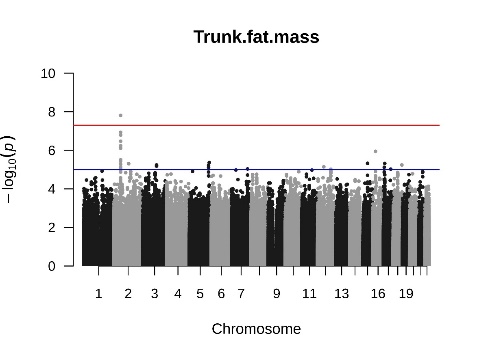
rs7592270 rs13212044 rs77772562**

**
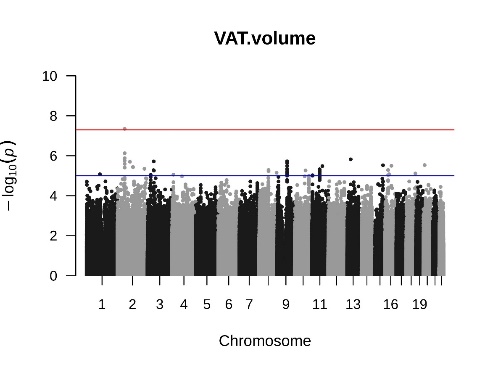

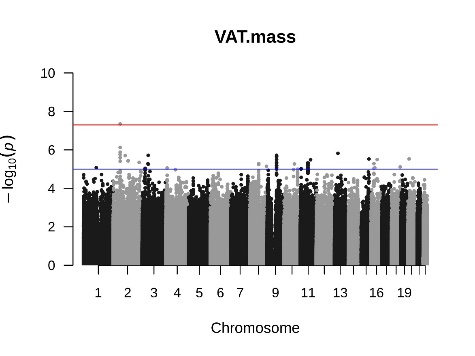

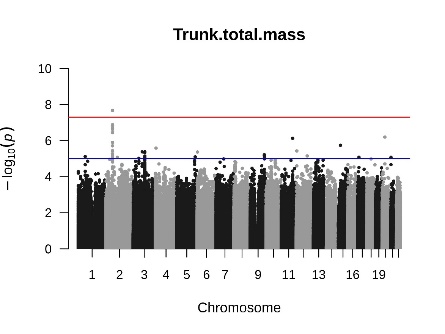

rs7592270 rs7592270 rs7592270**

**Figure S2.** Manhattan plot of SNPs statistically significant above line at p< 5.0 x 10^-8^ in the combined (male and female) GWAS (n=4 386; VAT n=4 336)

VAT, visceral adipose tissue

**rs117686994 rs35932350; rs117686994

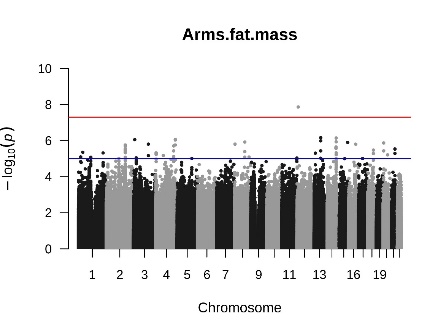

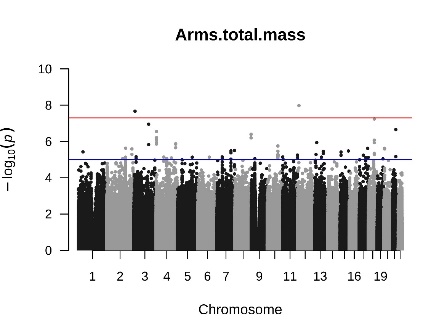
**

**rs112098641 rs2236705**

**
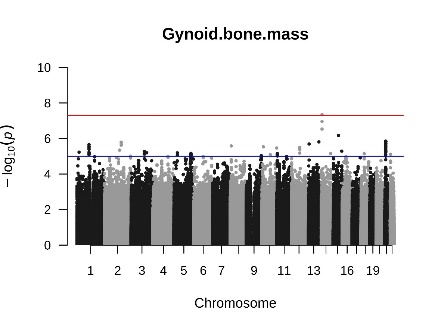

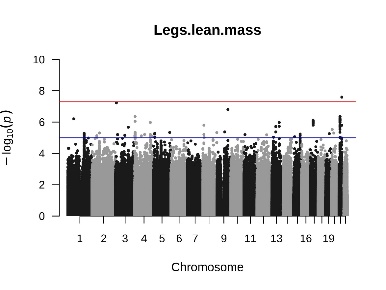
**

**rs164955; rs2236705 rs112098641**

**
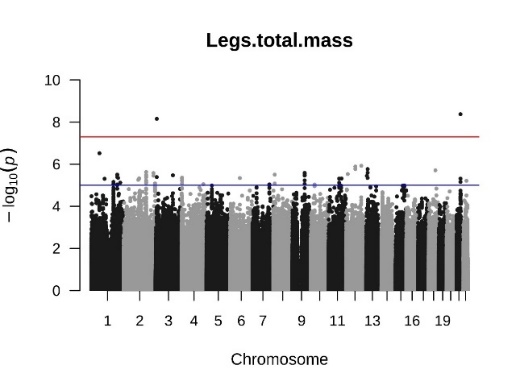

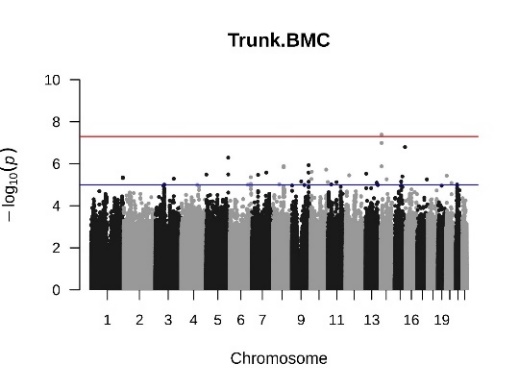
**

**Figure S3.** Manhattan plot of SNPs statistically significant above line at p<5.0 x 10^-8^ in the female only GWAS (n=2 294; VAT n=2 266)

BMC, bone mineral content


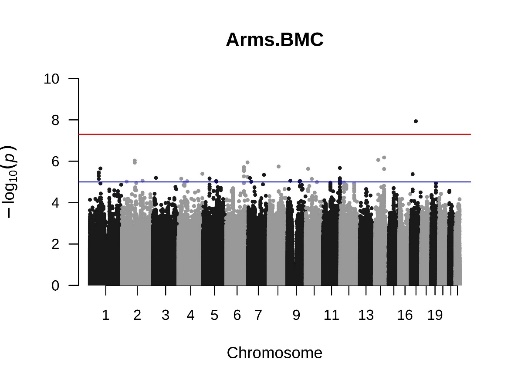
**rs138986597 rs55634776**


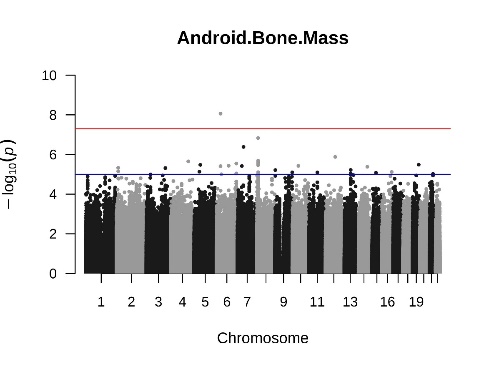


**rs72748040 rs145972737**

**
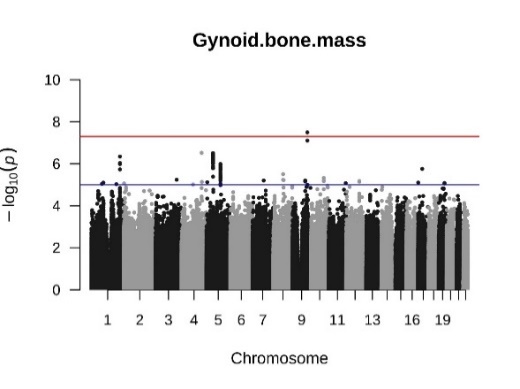

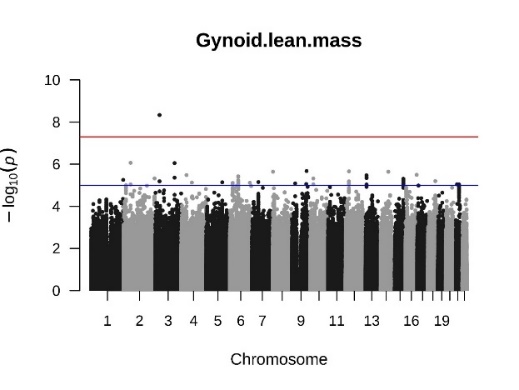
**

**rs113380185; 13:72584929_AAC_A rs143707182; rs138014219; 13:72584929_AAC_A**

**
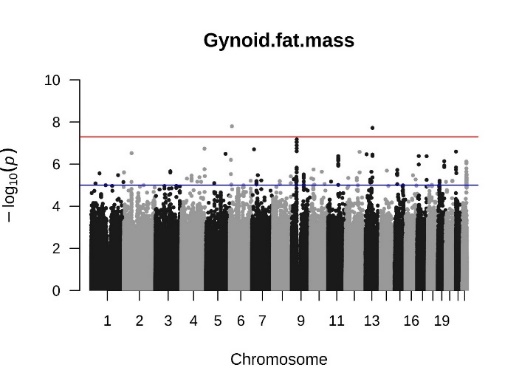

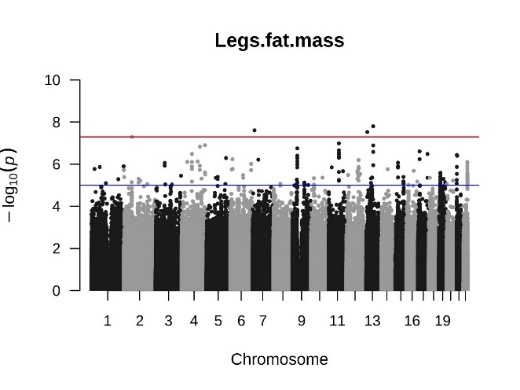
**

**rs7552312 rs1567843**

**
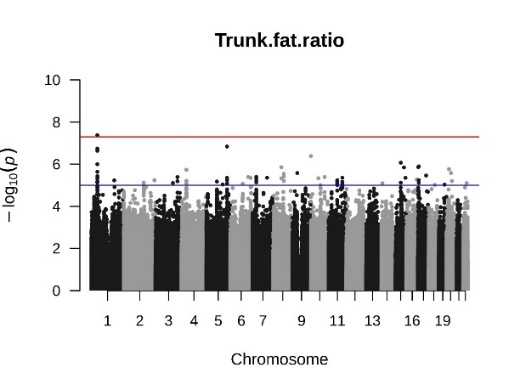
**
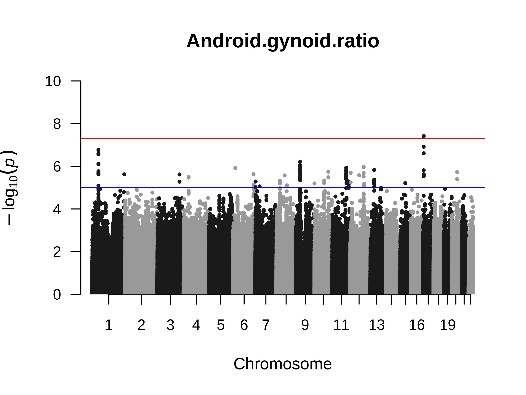


**Figure S4.** Manhattan plot of SNPs statistically significant above line at p< 5.0 x 10^-8^ in the male only GWAS (n=2 109; VAT n=2 088)

BMC, bone mineral content
